# Long‐Term Observational Results from the ASPIRE Study: OnabotulinumtoxinA Treatment for Adult Lower Limb Spasticity

**DOI:** 10.1002/pmrj.12517

**Published:** 2021-01-11

**Authors:** Alberto Esquenazi, Ganesh Bavikatte, Daniel S. Bandari, Wolfgang H. Jost, Michael C. Munin, Simon Fuk Tan Tang, Joan Largent, Aubrey Manack Adams, Aleksej Zuzek, Gerard E. Francisco

**Affiliations:** ^1^ MossRehab Gait and Motion Analysis Laboratory Elkins Park PA USA; ^2^ The Walton Centre Liverpool UK; ^3^ Multiple Sclerosis Center of California Laguna Hills CA USA; ^4^ Department of Neurology University of Freiburg Freiburg im Breisgau Germany; ^5^ Parkinson‐Klinik Ortenau Wolfach Germany; ^6^ Department of Physical Medicine and Rehabilitation University of Pittsburgh School of Medicine Pittsburgh PA USA; ^7^ Department of Physical Medicine and Rehabilitation Lotung Poh‐Ai Hospital Yilan Taiwan; ^8^ IQVIA Real‐World Evidence Solutions Cambridge MA USA; ^9^ Allergan, An AbbVie company Irvine CA USA; ^10^ University of Texas Health Science Center McGovern Medical School and TIRR Memorial Hermann Houston TX USA

## Abstract

**Introduction:**

OnabotulinumtoxinA treatment for spasticity varies according to numerous factors and is individualized to meet treatment goals.

**Objective:**

To explore real‐world onabotulinumtoxinA utilization and effectiveness in patients with lower limb spasticity from the Adult Spasticity International Registry (ASPIRE) study.

**Design:**

Two‐year, multicenter, prospective, observational registry (NCT01930786).

**Setting:**

Fifty‐four international clinical sites.

**Patients:**

Adults (naïve or non‐naïve to botulinum toxin[s] treatment for spasticity, across multiple etiologies) with lower limb spasticity related to upper motor neuron syndrome.

**Interventions:**

OnabotulinumtoxinA administered at the clinician's discretion.

**Main Outcome Measures:**

OnabotulinumtoxinA treatment utilization, clinician‐ and patient‐reported satisfaction.

**Results:**

In ASPIRE, 530 patients received ≥1 onabotulinumtoxinA treatment for lower limb spasticity (mean age, 52 years; stroke, 49.4%; multiple sclerosis, 20.4%). Equinovarus foot was treated most often (80.9% of patients), followed by flexed knee (26.0%), stiff extended knee (22.5%), and flexed toes (22.3%). OnabotulinumtoxinA doses ranged between 10 and 1100 U across all presentations. Electromyography (EMG) was most commonly used for injection localization (≥41.1% of treatment sessions). Despite low patient response on the satisfaction questionnaire, clinicians (94.6% of treatment sessions) and patients (84.5%) reported satisfaction/extreme satisfaction that treatment helped manage spasticity, and clinicians (98.3%) and patients (91.6%) would probably/definitely continue onabotulinumtoxinA treatment. These data should be interpreted with care. Twenty‐one adverse events (AEs) in 18 patients (3.4%) were considered treatment‐related. Sixty‐seven patients (12.6%) reported 138 serious AEs; 3 serious AEs in two patients (0.4%) were considered treatment‐related. No new safety signals were identified.

**Conclusions:**

ASPIRE provides long‐term observational data on the treatment of lower limb spasticity with onabotulinumtoxinA. Real‐world data from this primary analysis can help to guide the clinical use of onabotulinumtoxinA to improve spasticity management.

## Introduction

Spasticity is a chronic condition associated with several central nervous system disorders, including cerebral palsy, multiple sclerosis, spinal cord injury, stroke, and traumatic brain injury, as well as neurodegenerative diseases.^1^, ^2^, ^3^ Spasticity is part of the upper motor neuron (UMN) syndrome and describes disordered sensorimotor control, resulting from a UMN lesion, presenting as intermittent or sustained involuntary activation of muscles.^4^, ^5^ Spasticity in lower limb muscles affects coordinated movement of the ankle, knee, and hip,^6^, ^7^ impacting active and passive function,^8^, ^9^, ^10^ resulting in abnormal postures that can greatly interfere with mobility and gait,^9^, ^11^, ^12^ and can lead to limb pain and falls.^2^, ^6^, ^13^ Spasticity can negatively impact a patient's health‐related quality of life, place additional burden on caregivers, and lead to productivity and economic losses.^14^, ^15^, ^16^, ^17^, ^18^

Clinical approaches to manage spasticity often aim to address symptoms, ameliorate function, improve quality of life, and prevent secondary complications.^3^, ^19^ Spasticity management should be tailored to the needs of each patient, with adjunct therapies often recommended to improve outcomes.^20^, ^21^ The management of spasticity often includes the use of orthotics, assistive devices for walking, oral medications, intrathecal baclofen, botulinum toxins, and/or procedures/surgeries (see reviews^1^, ^2^, ^3^, ^8^, ^10^, ^22^). OnabotulinumtoxinA (BOTOX, Allergan, an AbbVie Company, North Chicago, Illinois, USA) is a focal neuromodulator that causes muscle relaxation by blocking acetylcholine at neuromuscular junctions^23^ and is approved worldwide for the management of adult upper and lower limb spasticity.^24^

The safety and efficacy of onabotulinumtoxinA for lower limb spasticity has been established in controlled clinical trials (eg,^25^, ^26^, ^27^, ^28^, ^29^, ^30^, ^31^, ^32^, ^33^, ^34^ and see reviews^8^, ^35^, ^36^). However, published real‐world data on the treatment of lower limb spasticity with onabotulinumtoxinA are limited, but are recommended to help guide clinical strategies to improve patient care.^8^, ^22^, ^37^ The Adult SPasticity International REgistry (ASPIRE) study was developed to describe the clinical characteristics of patients treated with onabotulinumtoxinA for spasticity and its burden across several etiologies and geographical regions over a 2‐year period.^38^ The main objectives of the ASPIRE study were to examine the patterns of onabotulinumtoxinA utilization and assess the effectiveness of onabotulinumtoxinA treatment for spasticity. A recent publication by Francisco et al^39^ described the clinical use of onabotulinumtoxinA for the treatment of adult upper limb spasticity from the ASPIRE study. Thus we feel it is beneficial to publish a complimentary manuscript focused on the lower limb population from ASPIRE, as many factors are likely to differ between the two spasticity populations, such as treatment goals (eg, walking [active function] vs. hygiene/self‐care activities [passive function]^40^, ^41^), patient underlying etiology (eg, patients with multiple sclerosis were treated for lower limb clinical presentations more often than other etiologies^42^), clinical presentations treated,^43^, ^44^ and onabotulinumtoxinA utilization approaches (eg, dosing likely to be impacted by larger muscles in the lower limb^43^). To this end, the current study evaluated the primary objectives of ASPIRE in patients treated for lower limb spasticity, defined as any enrolled patient who received ≥1 treatment of onabotulinumtoxinA to the lower limb during the study period.

## Methods

The ASPIRE study methods have been described previously in detail^38^, ^39^, ^42^ and are summarized in brief below.

### Study Design and Setting

ASPIRE is an international (Asia, Europe, and North America), multicenter (54 clinical sites, with 74 treating clinicians), prospective, observational registry (NCT01930786). OnabotulinumtoxinA treatments were given at the clinician's discretion in agreement with standard clinical practices and country‐specific regulations without intervention from the study sponsor. Re‐treatment with onabotulinumtoxinA was expected to occur approximately every 12 weeks.^24^, ^45^ Financial support was not provided for any treatment/treatment‐related costs. The ASPIRE study spanned 108 weeks: 96‐week study period and 12‐week follow‐up period. A study “completer” was defined as a patient who met all of the following criteria: (1) did not discontinue within the 96‐week study period, (2) was not lost to follow‐up, and (3) completed the Final Assessment form. Patients who did not meet all these criteria were labeled a “discontinuer.” ASPIRE was conducted in accordance with all applicable laws and regulations, including but not limited to, the Declaration of Helsinki and the Guidelines for Good Pharmacoepidemiology Practices (International Society for Pharmacoepidemiology [IPSE]).

### Participants

Included in the study were adults treated with onabotulinumtoxinA during the course of routine clinical care for spasticity related to UMN syndrome, regardless of previous exposure to botulinum toxin(s) for spasticity (naïve and non‐naïve). A full list of inclusion and exclusion criteria are provided in Francisco et al.^38^ Written informed consent was required for all patients. Each participating site obtained institutional review board approval.

### Outcomes and Data Sources

The primary objectives of ASPIRE were to (1) determine the patterns of utilization of onabotulinumtoxinA as a treatment for spasticity in clinical practice, and (2) quantify the effectiveness of onabotulinumtoxinA for the treatment of spasticity in clinical practice using clinician‐ and patient‐reported satisfaction. OnabotulinumtoxinA utilization was collected at each treatment session. Following treatment, clinician (each subsequent treatment session) and patient (5 ± 1 weeks post‐treatment) satisfaction data were collected.

Secondary objectives included: (1) patient‐reported outcome (PRO) data to evaluate the impacts of spasticity on quality of life, physical function, activities of daily life, and pain, and (2) estimation of the incidence of adverse events (AEs). In addition to satisfaction, the following patient‐ and clinician‐reported outcomes were gathered: Numeric Pain Rating Scale (NPRS^46^, ^47^), which was patient‐reported at baseline and 5 ± 1 weeks post‐treatment, and the Disability Assessment Scale (DAS^48^), which was clinician‐reported at treatment session 1 (prior to onabotulinumtoxinA administration) and at each subsequent treatment session. Safety data include any AE reported by patients from the lower limb population during the 108‐week study and considers total body dosing. AEs were summarized using the Medical Dictionary for Regulatory Activities (MedDRA) version 20.0 by system organ class and preferred term. Relationship to onabotulinumtoxinA treatment and evaluation of potential distant spread of toxin were adjudicated by a panel of safety clinicians. (Refer to Francisco et al^38^ for the complete data collection schedule.)

### Control for Bias

To minimize selection bias, broad eligibility criteria and a predetermined ratio of patients that were non‐naïve or naïve to botulinum toxin(s) for spasticity were utilized to ensure high generalizability to clinical practice. To minimize information bias and ensure data quality, the case report forms utilized in ASPIRE were carefully designed and training was provided to site staff. Clinicians were not compensated outside of registry administrative costs.

### Study Size, Statistical Methods, and Analysis Populations

Descriptive analyses of the study objectives did not test specific hypotheses, and therefore, no statistical power/sample size calculations were performed. Observed data are shown, with no imputation of missing values. Data collected outside of the 108‐week follow‐up period were not included. Statistical significance was determined using paired *t*‐tests with Bonferroni correction for NPRS and mixed ordinal logistic regression for DAS (Glimmix procedure) using SAS (version 9.2 or higher; SAS Institute, Cary, NC, USA).

The total analysis population (ie, all enrolled patients who received ≥1 treatment of onabotulinumtoxinA during the study) included the upper limb spasticity population^39^ and the lower limb spasticity population. For the lower limb population analysis, all enrolled patients (naïve or non‐naïve to botulinum toxin[s] for spasticity) who received ≥1 treatment of onabotulinumtoxinA to the lower limb during the study period were included. Notably, patients in the lower limb population may have also received treatment to the upper limb; however, only lower limb data are summarized in this manuscript.

## Results

### Patient Disposition

ASPIRE (dates: October 16, 2013 to October 9, 2017) enrolled 744 patients; 14 patients were excluded from the total analysis population (N = 14/744, 1.9%; Figure S1) and 730 patients were included (N = 730/744, 98.1%). During the 2‐year study, 530 patients received ≥1 treatment to the lower limb with onabotulinumtoxinA. Patients who were treated for upper limb spasticity only (N = 200) were not included in this analysis. Of the lower limb population (N = 530), 320 patients (60.4%) completed the ASPIRE study and 210 patients (39.6%) discontinued participation. Baseline demographics for completers and discontinuers are shown in Table S1. Of those that discontinued participation, 120 patients (57.1%) withdrew consent, 69 patients (32.9%) did not complete the Final Assessment form, and 21 patients (10.0%) were lost to follow‐up. The most commonly reported reason for withdrawal of consent was treatment ineffectiveness (N = 53/530, 10.0%). A complete list of reasons for study discontinuation is provided in Table S2.

### Demographics and Clinical Characteristics

At baseline, lower limb patients were on average 52 years old, 76.8% Caucasian (N = 407/530), 53.0% female (N = 281/530), and 36.4% (N = 193/530) naïve to botulinum toxin(s) for spasticity (Table 1). Baseline demographics for the lower limb population were similar to those observed in the total^38^ and upper limb^39^ populations. For primary underlying etiology, stroke was reported as the most prevalent diagnosis (N = 262/530, 49.4%), followed by multiple sclerosis (N = 108/530, 20.4%; Figure S2). Modified Modified Ashworth Scale^49^ severity scores revealed that most patients (N = 400/515, 77.7%) had either more marked or considerable increase in tone (Figure S3).

**Table 1:** Baseline Patient Demographics for the Lower Limb Population in the ASPIRE Study

	(N = 530)
Age (years)	
Mean (SD)	52.0 (15.4)
Median	53.0
Min, Max	18.5, 88.5
Gender, N (%)	
Female	281 (53.0)
Male	249 (47.0)
Race, N (%)	
Caucasian	407 (76.8)
Black/African/Caribbean	59 (11.1)
Asian	36 (6.8)
Latino/Hispanic	10 (1.9)
Middle Eastern/Arab	3 (0.6)
American Indian/Alaska Native	1 (0.2)
Other	3 (0.6)
Data Not Available	11 (2.1)
BMI (kg/m^2^), N	449
Mean (SD)	26.4 (5.4)
Median	25.5
Min, Max	16.5, 50.2
Naïve to botulinum toxin(s) for spasticity, N (%)	
Yes	193 (36.4)

BMI = body mass index; Max = maximum; Min = minimum; N = number of patients, SD = standard deviation.

### OnabotulinumtoxinA Treatment Utilization

**Figure 1:**
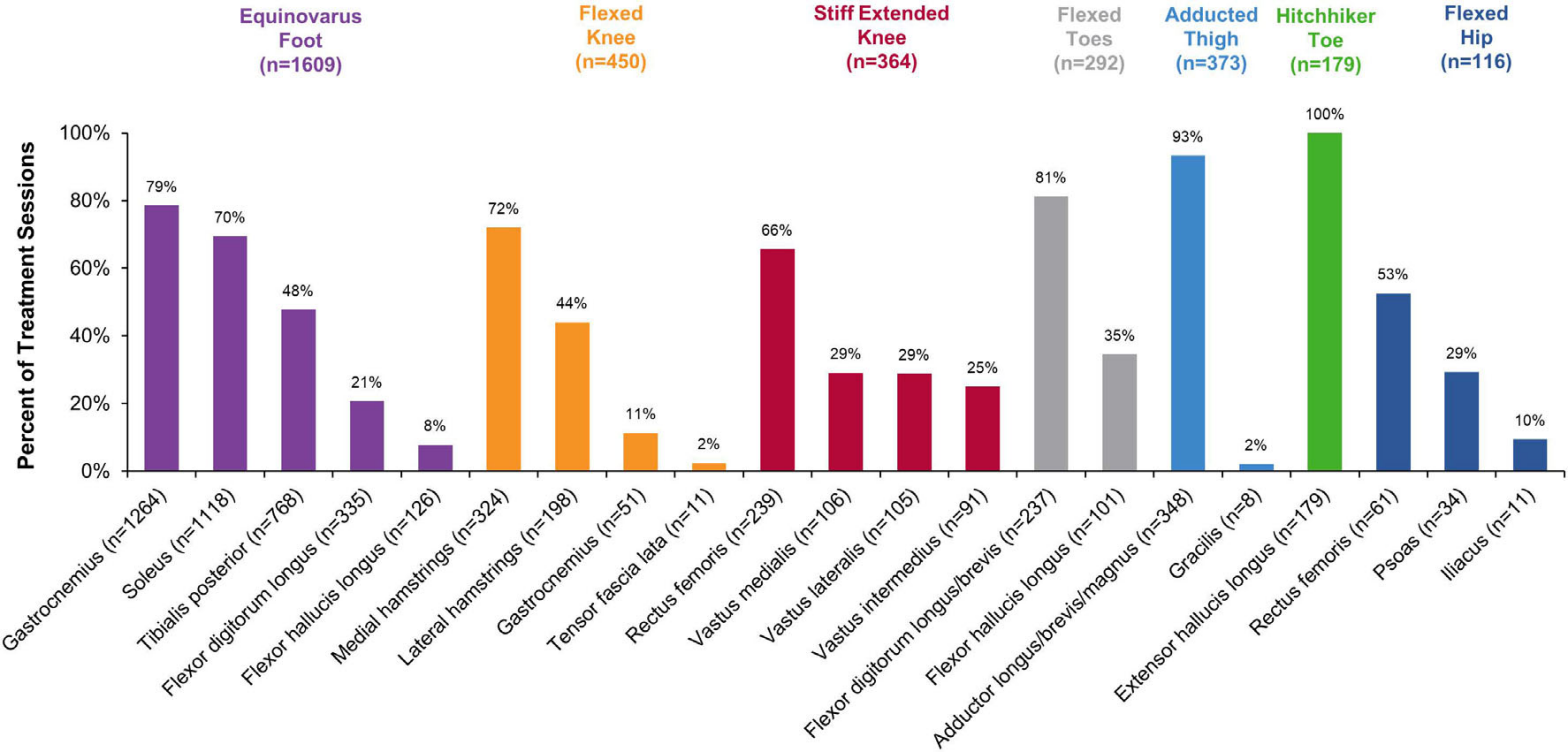
Muscles injected with onabotulinumtoxinA for the treatment of spasticity in the lower limb population. Data are stratified by clinical presentations and listed in order from highest number of patients treated to lowest. Lower limb presentations, and muscles for each clinical presentation, are not mutually exclusive. Therefore, the data shown may exceed 100%. Data for “other” presentations and “other” muscles that were not predefined in the case report form are not shown in the figure. n, number of treatment sessions for each clinical presentation or muscle injected.

During the 2‐year ASPIRE study, onabotulinumtoxinA was administered to the lower limb population (N = 530) in a total of 2105 treatment sessions. Across all sessions, the mean (standard deviation [SD]) treatment interval was 17.1 (7.2) weeks. In ASPIRE, the most commonly treated lower limb clinical presentations (in rank order by number of patients) were equinovarus foot, flexed knee, stiff extended knee, flexed toes, adducted thigh, striatal/hyperextended/hitchhiker toe, and flexed hip (refer to^43^, ^50^ for descriptions of each presentation). Data in Table 2 and Figure 1 show the variability and individualized nature of real‐world onabotulinumtoxinA utilization, with findings of interest highlighted below. For each clinical presentation, data shown are specific to that presentation only and do not represent an aggregate of all presentations treated at a given treatment session.

**Table 2:** Utilization of OnabotulinumtoxinA in Patients Treated for Lower Limb Spasticity (N = 530) in the ASPIRE Study^*^

	Equinovarus Foot	Flexed Knee	Stiff Ext. Knee	Flexed Toes	Adducted Thigh	Hitchhiker Toe	Flexed Hip
Patients, N (%)	429 (80.9)	138 (26.0)	119 (22.5)	118 (22.3)	107 (20.2)	65 (12.3)	44 (8.3)
Treatment Sessions, n	1609	450	364	292	373	179	116
Dose (U)							
Mean (SD)	220 (131)	154 (103)	138 (123)	68 (54)	162 (101)	43 (23)	93 (66)
Mode	200	100	100	50	100	50	100
Min, Max	15, 900	12, 1000	24, 1100	10, 400	20, 550	10, 100	15, 400
Dilution (U/mL),^†^ n (%)							
<25	55 ( 3.4)	16 ( 3.6)	10 ( 2.7)	2 ( 0.7)	9 ( 2.4)	5 ( 2.8)	4 ( 3.4)
25	236 (14.7)	50 (11.1)	28 ( 7.7)	17 ( 5.8)	55 (14.7)	29 (16.2)	17 (14.7)
50	667 (41.5)	159 (35.3)	182 (50.0)	142 (48.6)	137 (36.7)	72 (40.2)	54 (46.6)
100	577 (35.9)	182 (40.4)	114 (31.3)	117 (40.1)	135 (36.2)	69 (38.5)	23 (19.8)
Other	109 ( 6.8)	47 (10.4)	32 ( 8.8)	14 ( 4.8)	40 (10.7)	4 ( 2.2)	18 (15.5)
Needle Length (mm),^†^ n (%)							
10	111 ( 6.9)	31 ( 6.9)	7 ( 1.9)	29 ( 9.9)	21 ( 5.6)	20 (11.2)	0 ( 0.0)
37	743 (46.2)	162 (36.0)	121 (33.2)	120 (41.1)	113 (30.3)	83 (46.4)	49 (42.2)
50	407 (25.3)	95 (21.1)	113 (31.0)	82 (28.1)	75 (20.1)	46 (25.7)	23 (19.8)
75	34 ( 2.1)	2 ( 0.4)	9 ( 2.5)	5 ( 1.7)	1 ( 0.3)	1 ( 0.6)	2 ( 1.7)
Other	438 (27.2)	164 (36.4)	114 (31.3)	57 (19.5)	164 (44.0)	29 (16.2)	44 (37.9)
Injections,^†^ n (%)							
1	86 ( 5.3)	37 ( 8.2)	61 (16.8)	130 (44.5)	31 ( 8.3)	129 (72.1)	42 (36.2)
2	172 (10.7)	93 (20.7)	104 (28.6)	87 (29.8)	63 (16.9)	44 (24.6)	29 (25.0)
3	208 (12.9)	36 ( 8.0)	54 (14.8)	28 ( 9.6)	37 ( 9.9)	4 ( 2.2)	8 ( 6.9)
4	263 (16.3)	108 (24.0)	47 (12.9)	20 ( 6.8)	109 (29.2)	2 ( 1.1)	24 (20.7)
5	176 (10.9)	34 ( 7.6)	8 ( 2.2)	8 ( 2.7)	19 ( 5.1)	0 ( 0.0)	0 ( 0.0)
≥6	704 (43.8)	142 (31.6)	90 (24.7)	19 ( 6.5)	114 (30.5)	0 ( 0.0)	13 (15.5)
Treatment Side,^†^ n (%)							
Right	633 (39.3)	125 (27.8)	131 (36.0)	114 (39.0)	60 (16.1)	80 (44.7)	36 (31.0)
Left	708 (44.0)	131 (29.1)	121 (33.2)	161 (55.1)	70 (20.6)	91 (50.8)	33 (28.4)
Both	268 (16.7)	194 (43.1)	112 (30.8)	17 ( 5.8)	236 (63.3)	8 ( 4.5)	47 (40.5)
Localization Method,^‡^ n (%)							
Anatomical	556 (34.6)	212 (47.1)	126 (34.6)	120 (41.1)	213 (57.1)	45 (25.1)	51 (44.0)
E‐stim	435 (27.0)	26 ( 5.8)	79 (21.7)	118 (40.4)	36 ( 9.7)	63 (35.2)	6 ( 5.2)
EMG	807 (50.2)	254 (56.4)	175 (48.1)	147 (50.3)	153 (41.1)	74 (41.3)	68 (58.6)
Ultrasound	398 (24.7)	54 (12.0)	74 (20.3)	55 (18.8)	42 (11.3)	57 (31.8)	15 (12.9)

EMG = electromyography; E‐stim = electrical stimulation; Max = maximum; Min = minimum; N = number of patients; n = number of treatment sessions; SD = standard deviation; U = units of onabotulinumtoxinA.

^*^ Data are stratified by lower limb clinical presentations. Presentations and muscles targeted are not mutually exclusive, and therefore, may exceed 100%.

^†^ For each clinical presentation, data are the aggregate of all treatment sessions during the 2‐year study. Categories for dilution and needle length are not mutually exclusive.

^‡^ Injection localization methods were not mutually exclusive. Localization method data may not necessarily reflect clinician preference, but instead be an indication of equipment available at the study site. “Anatomical” localization refers to palpation. For each clinical presentation, data are the aggregate of all treatment sessions during the 2‐year study.

#### Equinovarus Foot

In 1609 treatment sessions, 429 patients (80.9%) received onabotulinumtoxinA for equinovarus foot (Table 2). To treat equinovarus foot, 200 U of onabotulinumtoxinA/session was utilized most often (mode). Of the available injection guidance techniques, clinicians commonly used electromyography (EMG) to locate the injection site(s) (n = 807/1609, 50.2%). OnabotulinumtoxinA was injected into the gastrocnemius (n = 1264/1609, 78.6%) and the soleus (n = 1118/1609, 69.5%; Figure 1) for most treatment sessions.

#### Flexed Knee

In 450 treatment sessions, 138 patients (26.0%) received onabotulinumtoxinA for flexed knee (Table 2). To treat flexed knee, 100 U of onabotulinumtoxinA/session was utilized most often. Clinicians commonly used EMG to locate injection site(s) (n = 254/450, 56.4%). OnabotulinumtoxinA was injected into the medial hamstrings for most treatment sessions (n = 324/450, 72.0%; Figure 1).

#### Stiff Extended Knee

In 364 treatment sessions, 119 patients (22.5%) received onabotulinumtoxinA for stiff extended knee (Table 2). To treat stiff extended knee, 100 U of onabotulinumtoxinA/session was utilized most often. Clinicians commonly used EMG to locate injection site(s) (n = 175/364, 48.1%). OnabotulinumtoxinA was injected into the rectus femoris for most treatment sessions (n = 239/364, 65.7%; Figure 1).

#### Flexed Toes

In 292 treatment sessions, 118 patients (22.3%) received onabotulinumtoxinA for flexed toes (Table 2). To treat flexed toes, 50 U of onabotulinumtoxinA/session was utilized most often. Clinicians commonly used EMG to locate injection site(s) (n = 147/292, 50.3%). OnabotulinumtoxinA was injected into the flexor digitorum longus/brevis for most treatment sessions (n = 237/292, 81.2%; Figure 1).

#### Adducted Thigh

In 373 treatment sessions, 107 patients (20.2%) received onabotulinumtoxinA for adducted thigh (Table 2). To treat adducted thigh, 100 U of onabotulinumtoxinA/session was utilized most often. Clinicians commonly used anatomical methods to locate injection site(s) (n = 213/373, 57.1%). OnabotulinumtoxinA was injected into the adductor longus/brevis/magnus for most treatment sessions (n = 348/373, 93.3%; Figure 1).

#### Striatal/Hyperextended/Hitchhiker Toe

In 79 treatment sessions, 65 patients (12.3%) received onabotulinumtoxinA for hitchhiker toe (Table 2). To treat hitchhiker toe, 50 U of onabotulinumtoxinA/session was utilized most often. Clinicians commonly used EMG to locate injection site(s) (n = 74/179, 41.3%). OnabotulinumtoxinA was injected into the extensor hallucis longus at every treatment session (n = 179/179, 100.0%; Figure 1).

#### Flexed Hip

In 116 treatment sessions, 44 patients (8.3%) received onabotulinumtoxinA for flexed hip (Table 2). To treat flexed hip, 100 U of onabotulinumtoxinA/session was utilized most often. Clinicians commonly used EMG to locate injection site(s) (n = 68/116, 58.6%). OnabotulinumtoxinA was injected into the rectus femoris for approximately half of treatment sessions (n = 61/116, 52.6%; Figure 1).

#### Adjustments to Muscles Targeted and OnabotulinumtoxinA Dose

**Figure 2:**
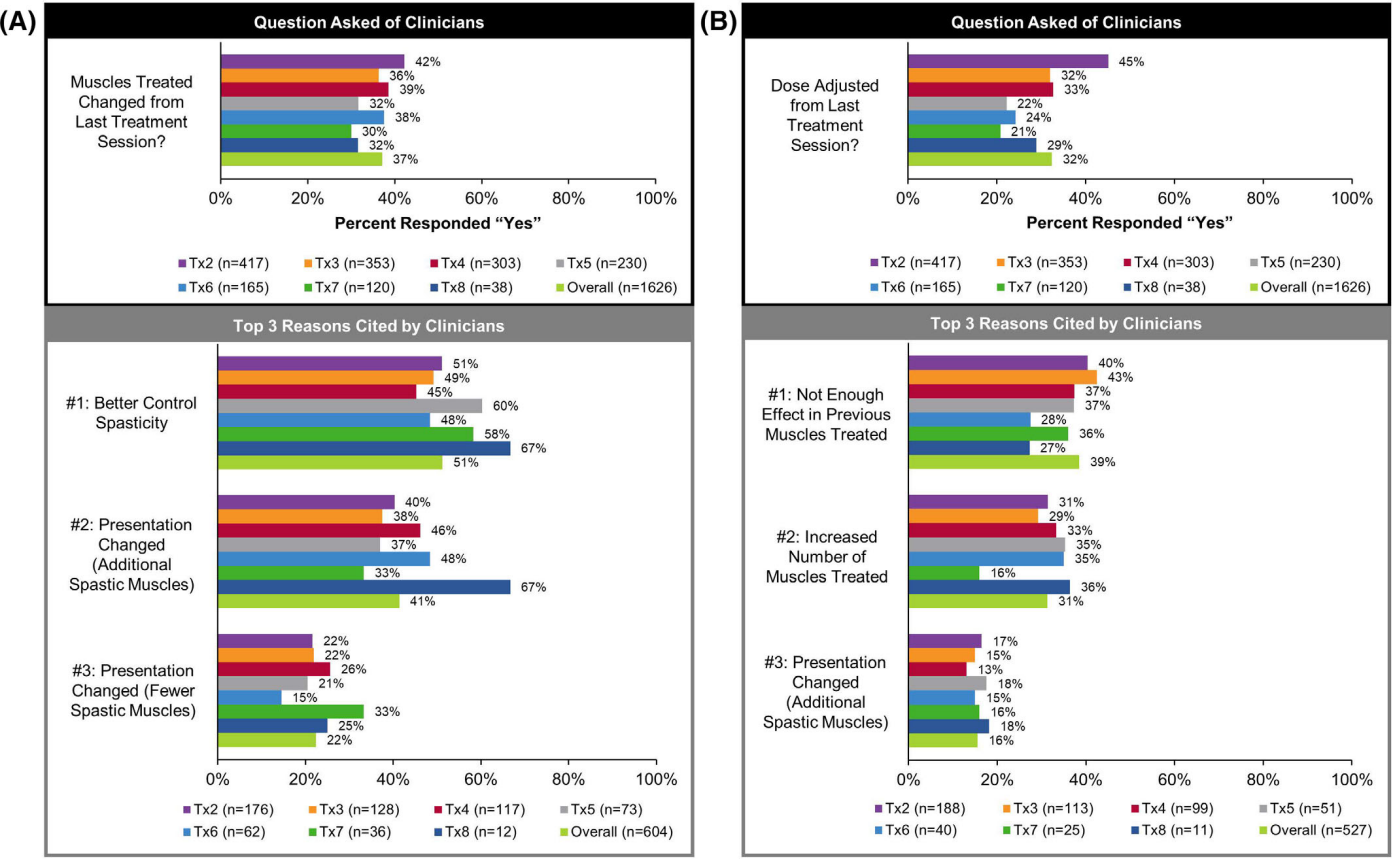
Adjustments to the muscles targeted and the dose of onabotulinumtoxinA utilized by clinicians at the time of re‐treatment in the lower limb population. At the time of re‐treatment, clinicians were asked whether (**A**) the muscles treated changed and (**B**) if the dose of onabotulinumtoxinA was adjusted from the last treatment session (shown in the black box). Of those that responded “yes” to the above questions, the three most common reasons cited by clinicians for this decision (excluding “other”) are provided in rank order (shown in the gray box). Clinicians could select more than one reason. n, number of treatment sessions; Tx, treatment session.

At the time of re‐treatment, clinicians were asked whether: (1) muscles treated changed and (2) dose was adjusted from the last treatment session (Figure 2). Clinicians adjusted the muscles treated in ~37% of treatment sessions (overall: n = 604/1612, 37.1%; Figure 2A), with the most common reason being “to better control spasticity” (overall: n = 309/604, 51.2%). Clinicians adjusted the dose of onabotulinumtoxinA in ~32% of treatment sessions (overall: n = 527/1626, 32.4%; Figure 2B), with the most common reason being “not enough effect in previous muscles treated” (overall: n = 203/527, 38.5%).

### Effectiveness

Several clinician‐ and patient‐reported outcomes were gathered in ASPIRE to evaluate the effectiveness of onabotulinumtoxinA to treat spasticity. However, patient response rates were low for NPRS and the patient satisfaction questionnaire. The number of responses at each treatment session for DAS, NPRS, and patient satisfaction, stratified by completers and discontinuers, is provided in Table 3.

**Table 3:** Proportion of Completers and Discontinuers who Responded to PRO Measures in the Lower Limb Population^*^

	Tx1	Tx2	Tx3	Tx4	Tx5	Tx6	Tx7	Tx8	Total
DAS, N^†^	529	465	394	336	257	184	128	39	2332
Completers, N (%)	320 (60.5)	316 (68.0)	298 (75.6)	272 (81.0)	224 (87.2)	171 (92.9)	122 (95.3)	37 (94.9)	1760 (75.5)
Discontinuers, N (%)	209 (39.5)	149 (32.0)	96 (24.4)	64 (19.0)	33 (12.8)	13 ( 7.1)	6 ( 4.7)	2 ( 5.1)	572 (24.5)
NPRS, N	140	155	169	161	146	101	76	25	1488
Completers, N (%)	87 (62.1)	109 (70.3)	127 (75.1)	129 (80.1)	130 (89.0)	94 (93.1)	73 (96.1)	25 (100.0)	1086 (73.0)
Discontinuers, N (%)	53 (37.9)	46 (29.7)	42 (24.9)	32 (19.9)	16 (11.0)	7 ( 6.9)	3 ( 3.9)	0 ( 0.0)	402 (27.0)
Patient Satisfaction, N^‡^	146	159	170	161	147	100	76	25	984
Completers, N (%)	89 (61.0)	111 (69.8)	127 (74.7)	129 (80.1)	131 (89.1)	93 (93.0)	73 (96.1)	25 (100.0)	778 (79.1)
Discontinuers, N (%)	57 (39.0)	48 (30.2)	43 (25.3)	32 (19.9)	16 (10.9)	7 ( 7.0)	3 ( 3.9)	0 ( 0.0)	206 (20.9)

DAS = Disability Assessment Scale; N = number of patients; NPRS = Numeric Pain Rating Scale; Tx = treatment session.

^*^ To be labeled a study “completer,” patients had to meet all of the following criteria: (1) did not discontinue within the 96‐week study period, (2) were not lost to follow‐up, and (3) completed the Final Assessment form. Any patient that did not meet all the criteria for a study completer, was labeled a study “discontinuer.”

^†^ The proportion of completers and discontinuers shown for DAS are representative of each subscale (dressing, hygiene, pain, posture, and mobility), as the sample size was the same for all subscales at each treatment session.

^‡^ The proportion of completers and discontinuers shown for the patient satisfaction questionnaire includes those that responded with “not applicable”. At each treatment session, the sample size was not the same for all nine items in the satisfaction questionnaire. To be conservative, the data shown in the table represent the lowest response rate observed at each treatment session.

#### Disability Assessment Scale (DAS)

OnabotulinumtoxinA treatment was followed by a significant improvement in DAS scores at subsequent treatment sessions, indicating a reduction in functional impairment over time, for the subscales of dressing, limb posture, mobility, and pain (all comparisons vs. treatment session 1, *P* < .0001; Table 4). The hygiene subscale did not improve significantly with onabotulinumtoxinA treatment.

**Table 4:** The Impact of OnabotulinumtoxinA Treatment for Spasticity on Disability Assessment Scale (DAS) Scores in the Lower Limb Population^*^

	Tx1 (N=529)^†^	Tx2 (N=465)	Tx3 (N=394)	Tx4 (N=336)	Tx5 (N=257)	Tx6 (N=184)	Tx7 (N=128)	Tx8 (N=39)
Dressing, N (%)								
0 ‐ No disability	90 (17.0)	92 (19.8)	70 (17.8)	64 (19.0)	53 (20.6)	46 (25.0)	28 (21.9)	7 (17.9)
1 ‐ Mild disability	172 (32.5)	173 (37.2)	158 (40.1)	136 (40.5)	103 (40.1)	83 (45.1)	52 (40.6)	17 (43.6)
2 ‐ Moderate disability	187 (35.3)	143 (30.8)	126 (32.0)	106 (31.5)	70 (27.2)	34 (18.5)	34 (26.6)	15 (38.5)
3 ‐ Severe disability	80 (15.1)	57 (12.3)	40 (10.2)	30 ( 8.9)	31 (12.1)	21 (11.4)	14 (10.9)	0 ( 0.0)
OR (95% CI)		1.7 (1.3, 2.3)	1.8 (1.3, 2.4)	2.0 (1.5, 2.8)	1.9 (1.3, 2.7)	2.5 (1.7, 3.8)	2.0 (1.3, 3.2)	2.9 (1.3, 6.1)
F‐Value: 4.7; *P* < .0001^‡^								
Hygiene, N (%)								
0 ‐ No disability	183 (34.6)	173 (37.2)	134 (34.0)	126 (37.5)	91 (35.4)	75 (40.8)	48 (37.5)	11 (28.2)
1 ‐ Mild disability	139 (26.3)	130 (28.0)	135 (34.3)	105 (31.3)	79 (30.7)	58 (31.5)	41 (32.0)	13 (33.3)
2 ‐ Moderate disability	142 (26.8)	115 (24.7)	90 (22.8)	79 (23.5)	59 (23.0)	31 (16.8)	27 (21.1)	15 (38.5)
3 ‐ Severe disability	65 (12.3)	47 (10.1)	35 ( 8.9)	26 ( 7.7)	28 (10.9)	20 (10.9)	12 ( 9.4)	0 ( 0.0)
OR (95% CI)		1.4 (1.0, 1.8)	1.3 (1.0, 1.8)	1.5 (1.1, 2.0)	1.0 (0.7, 1.5)	1.3 (0.9, 2.0)	1.3 (0.8, 2.2)	1.5 (0.7, 3.1)
F‐Value: 1.3; *P* = .2258								
Limb Posture, N (%)								
0 ‐ No disability	54 (10.2)	55 (11.8)	53 (13.5)	45 (13.4)	36 (14.0)	29 (15.8)	20 (15.6)	4 (10.3)
1 ‐ Mild disability	128 (24.2)	150 (32.3)	152 (38.6)	126 (37.5)	97 (37.7)	79 (42.9)	46 (35.9)	19 (48.7)
2 ‐ Moderate disability	242 (45.7)	202 (43.4)	146 (37.1)	125 (37.2)	95 (37.0)	57 (31.0)	46 (35.9)	13 (33.3)
3 ‐ Severe disability	105 (19.8)	58 (12.5)	43 (10.9)	40 (11.9)	29 (11.3)	19 (10.3)	16 (12.5)	3 ( 7.7)
OR (95% CI)		2.1 (1.6, 2.8)	3.1 (2.3, 4.2)	3.0 (2.2, 4.1)	3.0 (2.1, 4.2)	3.1 (2.1, 4.6)	2.5 (1.6, 3.9)	3.0 (1.4, 6.3)
F‐Value: 11.5; *P* < .0001								
Mobility, N (%)								
0 ‐ No disability	27 ( 5.1)	22 ( 4.7)	20 ( 5.1)	15 ( 4.5)	15 ( 5.8)	8 ( 4.3)	5 ( 3.9)	0 ( 0.0)
1 ‐ Mild disability	67 (12.7)	102 (21.9)	97 (24.6)	94 (28.0)	67 (26.1)	59 (32.1)	35 (27.3)	12 (30.8)
2 ‐ Moderate disability	262 (49.5)	235 (50.5)	191 (48.5)	161 (47.9)	129 (50.2)	86 (46.7)	58 (45.3)	21 (53.8)
3 ‐ Severe disability	173 (32.7)	106 (22.8)	86 (21.8)	66 (19.6)	46 (17.9)	31 (16.8)	30 (23.4)	6 (15.4)
OR (95% CI)		2.5 (1.9, 3.4)	2.9 (2.1, 4.0)	3.5 (2.5, 5.0)	4.0 (2.8, 5.8)	3.3 (2.2, 5.0)	2.5 (1.6, 4.1)	3.1 (1.4, 6.7)
F‐Value: 11.6; *P* < .0001								
Pain, N (%)								
0 ‐ No disability	188 (35.5)	196 (42.2)	182 (46.2)	139 (41.4)	113 (44.0)	88 (47.8)	55 (43.3)	18 (46.2)
1 ‐ Mild disability	138 (26.1)	135 (29.0)	108 (27.4)	112 (33.3)	75 (29.2)	48 (26.1)	34 (26.8)	11 (28.2)
2 ‐ Moderate disability	134 (25.3)	94 (20.2)	78 (19.8)	61 (18.2)	52 (20.2)	33 (17.9)	26 (20.5)	8 (20.5)
3 ‐ Severe disability	69 (13.0)	40 ( 8.6)	26 ( 6.6)	24 ( 7.1)	17 ( 6.6)	15 ( 8.2)	12 ( 9.4)	2 ( 5.1)
OR (95% CI)		2.0 (1.5, 2.6)	2.6 (1.9, 3.5)	2.2 (1.6, 3.0)	2.2 (1.6, 3.2)	2.4 (1.6, 3.7)	1.8 (1.1, 2.8)	2.9 (1.3, 6.3)
F‐Value: 6.8; *P* < .0001								

CI = confidence interval; N = number of patients; OR = odds ratio; Tx = treatment session.

^*^ The DAS was developed to objectively measure functional impairment resulting from spasticity across 5 subscales, including dressing, hygiene, limb posture, mobility, and pain.^48^ For each subscale, patients were evaluated on a 4‐point scale (range: 0‐3), where “0” represents no disability and “3” represents severe disability (normal activities limited). DAS was assessed by the clinician at treatment session 1 (prior to onabotulinumtoxinA administration) and at each subsequent treatment session.

^†^ For statistical analysis, data from treatment session 1 were used as a reference.

^‡^ To account for repeated measures (ie, each individual at the start of each treatment session), data were analyzed using a general linear mixed model (mixed ordinal logistic regression). The outcome consisted of ordinal categories; therefore, a multinomial distribution was used to perform ordinal logistic regression. For each subscale, the F value and level of significance (*P* value) are shown.

#### Numeric Pain Rating Scale (NPRS)

**Figure 3:**
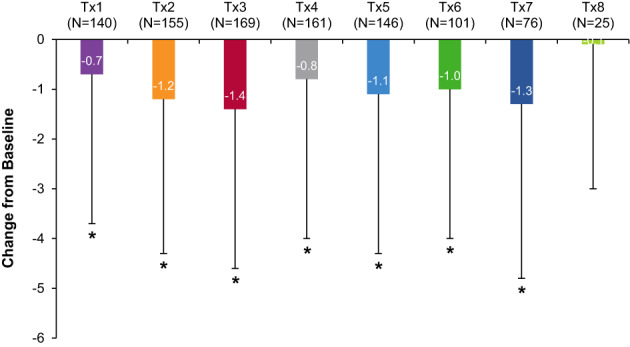
Numeric Pain Rating Scale (NPRS) following onabotulinumtoxinA treatment for spasticity in the lower limb population. NPRS is an 11‐point rating scale (range: 0 to 10), where “0” represents no pain and “10” represents the worst pain imaginable, that is used to assess pain intensity.^46^, ^47^ Patient‐reported NPRS data were gathered at baseline (prior to onabotulinumtoxinA treatment), as well as 5 ± 1 weeks post‐treatment via phone or web. The mean change in NPRS scores versus baseline are shown. *Indicates a statistically significant change from the baseline score at *P* < .006 (Bonferroni correction applied). N, number of patients; Tx, treatment session.

The NPRS score at baseline (N = 515) was 4.0 ± 3.2 (mean ± SD). Of those patients that completed the assessment (on average ~52% response rate across all treatment sessions; detailed in Table S3), onabotulinumtoxinA treatment was followed by a significant improvement in mean NPRS scores (range: −0.1 to −1.4; Figure 3), indicating a reduction in patient‐reported spasticity‐related pain (treatment sessions 1 to 7 vs. baseline; all *P* < .006).

#### Clinician Satisfaction

**Figure 4:**
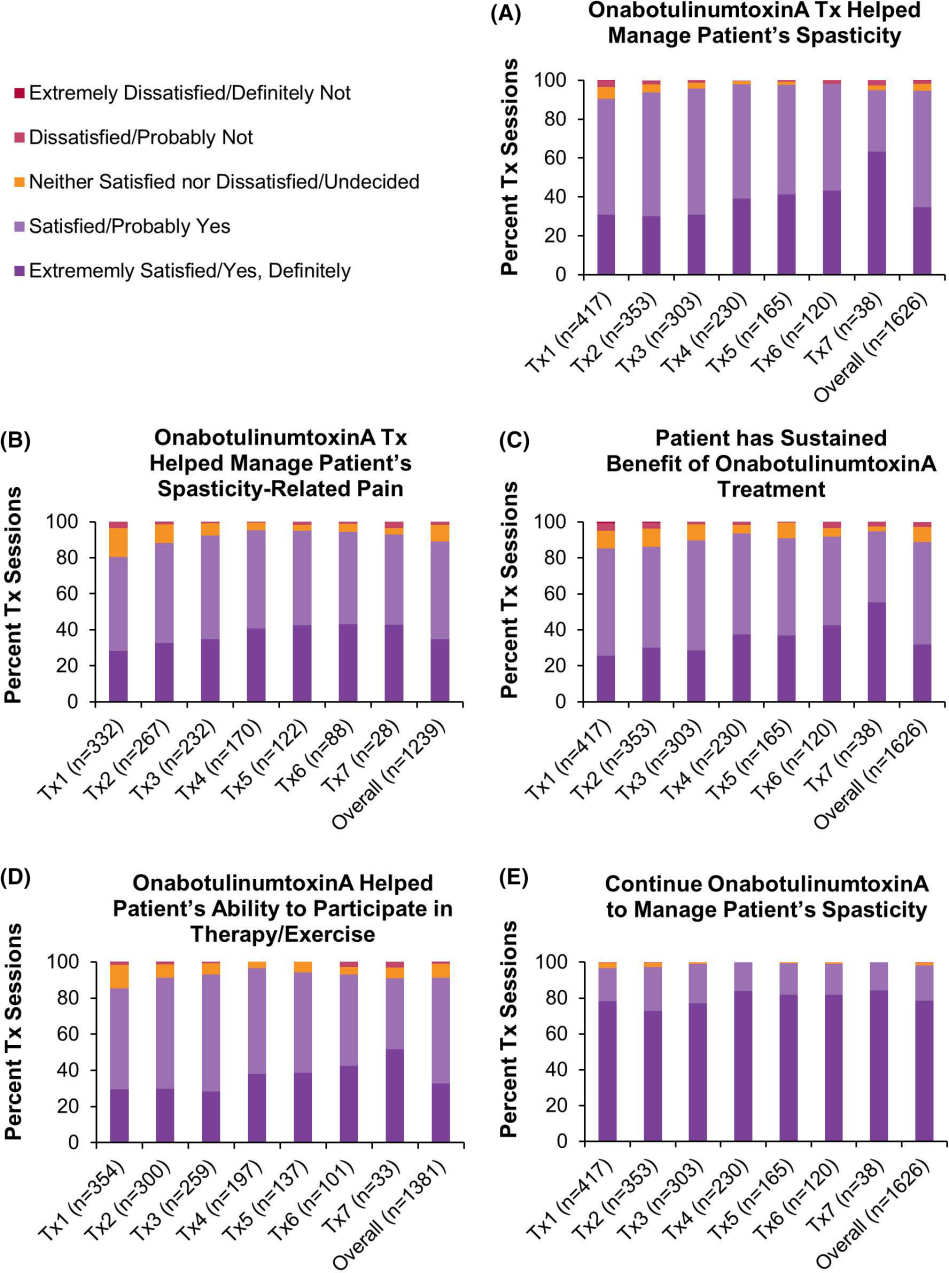
Clinician satisfaction with onabotulinumtoxinA for the treatment of spasticity in the lower limb population. At each subsequent treatment session, clinician satisfaction with the previous onabotulinumtoxinA (referred to as BOTOX in the case report form) treatment was collected. Consequently, data on clinician satisfaction at treatment session 8 and/or the final treatment session were not collected. For Figures (B) and (D), the percentage of treatment sessions were recalculated to exclude those in which clinicians indicated that the question was “not applicable.” Data are presented as percent of treatment sessions. n, number of treatment sessions; Tx, treatment session.

Of patients who were evaluated at subsequent treatment sessions, clinicians reported satisfaction/extreme satisfaction that onabotulinumtoxinA helped manage a patient's spasticity (overall: 94.6% of treatment sessions; Figure 4A) and had sustained benefit of treatment (overall: 84.3%; Figure 4C). Clinicians reported satisfaction/extreme satisfaction that onabotulinumtoxinA helped manage a patient's spasticity‐related pain (overall: 89.0%; Figure 4B) and helped patients participate in therapy/exercise (overall: 91.2%; Figure 4D). Clinicians responded that they would probably/definitely continue onabotulinumtoxinA to manage their patient's spasticity (overall: 98.3%; Figure 4E).

#### Patient Satisfaction

**Figure 5:**
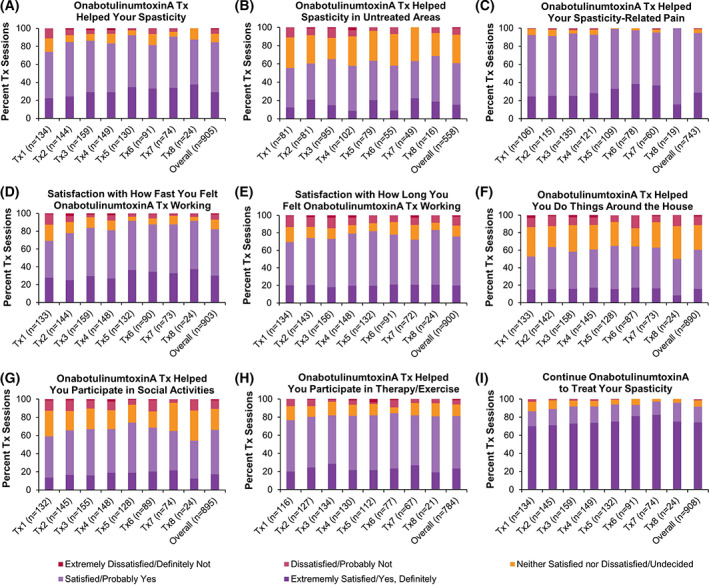
Patient satisfaction with onabotulinumtoxinA for the treatment of spasticity in the lower limb population. At 5 ± 1 weeks post‐treatment, patient satisfaction with onabotulinumtoxinA (referred to as BOTOX in the case report form) treatment was collected via phone or web. For Figures (B), (C), and (H), the percentage of treatment sessions were recalculated to exclude those in which patients indicated that the question was “not applicable.” Data are presented as percent of treatment sessions. n, number of treatment sessions; Tx, treatment session.

Of those patients that completed the questionnaire, most reported satisfaction/extreme satisfaction that onabotulinumtoxinA helped their spasticity (overall: 84.5% of treatment sessions; Figure 5A) and their spasticity‐related pain (overall: 84.9%; Figure 5C), as well as helped them participate in therapy/exercise (overall: 81.1%; Figure 5H). Patients were satisfied/extremely satisfied with how fast (overall: 82.3%; Figure 5D) and how long (overall: 75.7%; Figure 5E) they felt the onabotulinumtoxinA treatment working. Patients agreed that they would probably/definitely continue onabotulinumtoxinA to manage their spasticity (overall: 91.6%; Figure 5I). However, due to low patient response on the questionnaire (~40% of patients across all treatment sessions [detailed in Table S4]), these results should be interpreted with care.

### Safety and Tolerability

Throughout the 108‐week study, a total of 643 AEs were reported by 197 patients (37.2%; Table S5) in the lower limb population, with 21 events in 18 patients (3.4%) considered related to treatment (Table 5). Muscular weakness was the most frequently reported treatment‐related AE, with six events reported in six patients (1.1%). A total of 138 serious AEs were reported by 67 patients (12.6%; Table S5). Of the serious AEs reported, three events in two patients (0.4%) were considered related to treatment (Table 5). Specifically, one male patient with stroke had muscular weakness (thumb) and a second male patient with stroke had dysphagia and slow speech, all related to upper limb treatment. A panel of safety clinicians adjudicated that neither case was related to distant spread of toxin. An AE leading to study withdrawal occurred in six patients; two AEs were considered related to treatment and included drug tolerance and asthenia. In total, 13 deaths were reported during the study. Of these deaths, 10 were in the lower limb population; none of the deaths were considered related to treatment.

**Table 5:** Treatment‐Related Adverse Events and Treatment‐Related Serious Adverse Events Reported in the Lower Limb Population in the ASPIRE Study^*^

	Patients, N (%)	Events, n
TRAEs		
Muscular weakness	6 (1.1)	6
Asthenia	2 (0.4)	2
Dysphagia	2 (0.4)	2
Drug tolerance	1 (0.2)	1
Dry mouth	1 (0.2)	1
Fall	1 (0.2)	1
Gait disturbance	1 (0.2)	1
Grip strength decreased	1 (0.2)	1
Influenza‐like illness	1 (0.2)	1
Nausea	1 (0.2)	1
Peripheral edema	1 (0.2)	1
Slow speech	1 (0.2)	1
Vomiting	1 (0.2)	1
Weight increased	1 (0.2)	1
TRSAEs		
Dysphagia	1 (0.2)	1
Muscular weakness	1 (0.2)	1
Slow speech	1 (0.2)	1

n = number of adverse events; N = number of patients; TRAE = treatment‐related adverse events; TRSAE = treatment‐related serious adverse events.

^*^ All TRAE data and TRSAE data are provided in the table.

## Discussion

Data from controlled trials on the treatment of lower limb post‐stroke spasticity with onabotulinumtoxinA have been published previously (eg,^26^, ^28^, ^29^, ^30^, ^33^). However, the use of onabotulinumtoxinA to treat spasticity associated with other etiologies, as well as data from real‐world clinical practice, are necessary to guide clinical strategies to optimize patient care and improve clinician education. The goals of ASPIRE were to examine real‐world onabotulinumtoxinA treatment patterns, as well as to quantify the effectiveness of onabotulinumtoxinA for spasticity using clinician‐ and patient‐reported outcomes. ASPIRE is the largest international, observational registry examining onabotulinumtoxinA utilization for the treatment of spasticity across multiple etiologies and geographical regions. ASPIRE spanned 54 international sites in Asia, Europe, and North America. Data gathered from ASPIRE represent real‐world clinical practice, increasing its external validity and generalizability compared to previously published controlled trials. Specifically, this ASPIRE analysis describes the population treated for lower limb spasticity, which included patients that received ≥1 treatment with onabotulinumtoxinA to the lower limb during the 2‐year study.

In ASPIRE, patients were treated at the clinician's discretion, including muscles targeted, onabotulinumtoxinA dosing, targeting method(s), and time to retreatment. The study protocol did not dictate a predetermined number of treatment sessions. Given the conservative definition utilized for study completers in ASPIRE (ie, did not discontinue, were not lost to follow‐up, and completed the Final Assessment form), a patient could have received a single treatment during the 2‐year study (as prescribed by their clinician), fulfilling the criteria for “completer,” while another patient may have completed eight treatment sessions, but failed to complete the final assessment form, and was categorized as a “discontinuer.” Importantly, regardless of completer/discontinuer status, data presented in this manuscript include all observed data up until the time a patient discontinued the study or was lost to follow‐up.

Of the clinical presentations associated with lower limb spasticity,^7^, ^19^ equinovarus foot was treated most often in ASPIRE, as determined by rank order of patients treated per presentation across all treatment sessions. The second most common presentation was flexed knee, followed by stiff extended knee, flexed toes, adducted thigh, hitchhiker toe, and flexed hip. Variability in certain aspects of onabotulinumtoxinA utilization, such as needle length, number of injections, and onabotulinumtoxinA dilution, were observed. Differences in these factors are likely a reflection of the specific muscles/muscle groups being targeted for each presentation.^43^ Muscles targeted in ASPIRE were comparable to those described previously,^7^, ^10^, ^51^ and likely reflect similar approaches among clinicians to treat common spasticity presentations.

In this study, a large range of onabotulinumtoxinA doses were utilized across clinical presentations, with the highest dose range observed for stiff extended knee (minimum to maximum = 1076 U). Despite these large dose ranges, the mean dose of onabotulinumtoxinA for each lower limb presentation was within (or less than) that recommended by a recent Delphi panel.^43^ Conversely, the maximum dose of onabotulinumtoxinA reported for several presentations was higher than that recommended by the product label (ie, 400 U^24^), and can likely be attributed to the inherent complexities of treating patients in the real‐world.^19^, ^41^, ^43^ Dosing decisions are often influenced by patient condition, muscles targeted, potential for AEs, clinician experience, and if applicable, a patient's previous response to treatment.^19^, ^35^, ^52^ Indeed, when clinicians were asked whether they adjusted the dose of onabotulinumtoxinA from the previous treatment session, ~32% of clinicians indicated that they changed the dose due to not enough effect in previous muscles treated and ~ 37% of clinicians indicated that they adjusted the muscles targeted to better control spasticity. Altogether, utilization data from ASPIRE reflect the differences and similarities in approaches utilized by clinicians to treat lower limb spasticity and are consistent with published recommendations.^19^, ^20^, ^43^ These data affirm that spasticity management approaches are variable, individualized, and further highlight the need to continually reappraise treatment strategies to meet selected goals, while taking into consideration the potential for AEs.

To determine the effectiveness of onabotulinumtoxinA for the treatment of spasticity, ASPIRE collected data on clinician‐ and patient‐reported outcomes,^53^, ^54^ including satisfaction, functional impairment using DAS,^48^ and pain intensity using NPRS.^46^, ^47^ Certain forms of data can only be obtained from the patient, such as the impact of the disease or condition on daily living, making PRO data important and necessary.^53^ Satisfaction data gathered in ASPIRE indicate that most clinicians and patients who completed the questionnaire were satisfied with onabotulinumtoxinA to treat spasticity. This finding is supported by the observation that only 10% of patients discontinued the study due to treatment ineffectiveness. Most clinicians and patients were satisfied that onabotulinumtoxinA treatment helped participation in therapy/exercise. This is an important finding, as a multifaceted approach is recommended to best meet treatment goals.^20^, ^21^ Despite these favorable findings, interpretation of the patient satisfaction data is complicated by the reduced questionnaire response rate observed (~40% across all treatment sessions), and therefore, these results should be interpreted with care, as they may not reflect all patients that received onabotulinumtoxinA treatment in ASPIRE.

OnabotulinumtoxinA treatment significantly reduced DAS scores over time, demonstrating improved quality of life in patients with repeated, long‐term treatment. Specifically, significant improvements in dressing, limb posture, mobility, and pain were observed following onabotulinumtoxinA treatment, but not hygiene. The likely explanation for the lack of significance in hygiene is that most patients in ASPIRE were treated for equinovarus foot (~81%), for which hygiene is of lower concern than the other DAS subscales. Altogether, these findings are important, as diminished health‐related quality of life has been associated with increased disability on the DAS in patients with upper limb post‐stroke spasticity.^55^

Finally, of those patients who completed the NPRS, significant improvement in mean NPRS scores compared to baseline were reported following onabotulinumtoxinA, which indicates that patients self‐reported less spasticity‐related pain following treatment. Improvements in NPRS are further supported by patient‐reported satisfaction data indicating that most patients who completed the questionnaire were satisfied that onabotulinumtoxinA treatment helped manage their spasticity‐related pain. Combined, these data agree with previous findings demonstrating that onabotulinumtoxinA treatment can reduce spasticity‐related pain (eg,^26^, ^40^, ^56^, ^57^ and see review^58^), which when present, can lead to negative impacts on health‐related quality of life,^59^, ^60^ work productivity, and financial loss.^61^ Similar to the patient satisfaction data, these findings should be interpreted with care due to reduced response rates for NPRS (~52% of patients across all treatment sessions). Despite these limitations, the ability of onabotulinumtoxinA to reduce pain in patients with spasticity remains a valuable treatment outcome from ASPIRE.

The safety and tolerability of onabotulinumtoxinA treatment for lower limb spasticity has been demonstrated within the literature (see reviews^36^, ^62^, ^63^). Long‐term observational data from ASPIRE add to the body of evidence on the safety and tolerability of onabotulinumtoxinA treatment for adults with spasticity. Safety data from ASPIRE is inclusive of patients across several different etiologies, in those naïve or non‐naïve to botulinum toxin(s) for spasticity, with a wide range of doses, for a variety of lower limb clinical presentations, as well as several geographical regions and clinician specialties. OnabotulinumtoxinA demonstrated an acceptable safety profile, with no new safety signals identified. It is important to continuously monitor for safety and consider potential AEs when forming a treatment plan. Safety data captured in ASPIRE are consistent with data listed in the package insert^24^ and reported within the literature.^40^, ^63^

Limitations of the ASPIRE study have been discussed previously.^38^ The design of the ASPIRE study (large, observational registry) resulted in a lack of control over study elements and confounding factors. Data at later timepoints should be interpreted with caution due to lower sample size. Data reported by patients via phone or web (ie, patient satisfaction or NPRS) had much lower response rates compared to other measures, which may indicate that patients found these assessments difficult or burdensome to complete. Data obtained at subsequent treatment sessions, such as clinician satisfaction, may have been negatively impacted by patient discontinuation from the study. Patient discontinuation could also affect clinician‐ and patient‐reported measures, as we would predict that patients with favorable outcomes would be more likely to remain in the study. However, as shown in Table 3, both completers and discontinuers responded to DAS, NPRS, and patient satisfaction throughout all treatment sessions. Furthermore, assessments collected outside of the clinic (ie, NPRS and patient satisfaction) included patients discontinuing treatment/not planning to return for subsequent treatments. For this analysis of the lower limb population, patients may have been treated for the upper limb as well; therefore, these data are not exclusive to the lower limb, and may have been influenced by treatment to the upper limb, reflecting more holistic improvements (eg, PROs) or total body dosing (eg, AEs). At baseline, approximately one‐third of enrolled patients were naïve to botulinum toxin(s) for spasticity, in accordance with the ASPIRE study protocol.^38^ Based on the data shown in Table S1, treatment‐naïve patients were more likely to discontinue the study than non‐naïve patients, highlighting the need for clinicians to set clear goals and expectations at treatment onset with onabotulinumtoxinA‐naïve patients. As a result of study design and patient discontinuation, the data presented in this manuscript represent majority non‐naïve patients, which may have biased the patient population in favor of those in which onabotulinumtoxinA was tolerable and effective; however, inclusion of both naïve and non‐naïve patients is reflective of real‐world clinical practice. To further guide spasticity management strategies, future analyses from ASPIRE should explore the impact of treatment history (naïve vs. non‐naïve to botulinum toxin[s] for spasticity) and hemiplegia on onabotulinumtoxinA utilization and treatment outcomes.

## Conclusions

ASPIRE provides long‐term observational data on the treatment of adult lower limb spasticity with onabotulinumtoxinA over 2 years, across a variety of underlying etiologies and several geographical regions. ASPIRE demonstrated individualized onabotulinumtoxinA utilization for the treatment of lower limb spasticity. Real‐world data from this primary analysis, combined with previously published controlled trial data, can help to guide the clinical use of onabotulinumtoxinA to improve spasticity management programs.

## Data Sharing Statement

AbbVie is committed to responsible data sharing regarding the clinical trials we sponsor. This includes access to anonymized, individual, and trial‐level data (analysis data sets), as well as other information (eg, protocols and Clinical Study Reports), as long as the trials are not part of an ongoing or planned regulatory submission. This includes requests for clinical trial data for unlicensed products and indications. These clinical trial data can be requested by any qualified researchers who engage in rigorous, independent scientific research, and will be provided following review and approval of a research proposal and Statistical Analysis Plan (SAP) and execution of a Data Sharing Agreement (DSA). Data requests can be submitted at any time and the data will be accessible for 12 months, with possible extensions considered. For more information on the process, or to submit a request, visit the following link: https://www.abbvie.com/our‐science/clinical‐trials/clinical‐trials‐data‐and‐information‐sharing/data‐and‐information‐sharing‐with‐qualified‐researchers.html.

**CME Question**

In this study, the mean dose of Onabotulinumtoxin A for each lower limb presentation (equinovarus, flexed knee, stiff knee, flexed toes or extended big toe) was:

- Within or less than the ranges recommended by a previous Delphi panel in 2017
- Higher than the maximum dose recommended by the product label
- The same for different targeted muscles in the lower limb
- Similar at repeat injections across all clinical presentations

**Answer online at** https://onlinelearning.aapmr.org/

## Supporting information


**Appendix S1** Supporting information
