# Supplementary material for: Long‐Term Observational Results from the ASPIRE Study: OnabotulinumtoxinA Treatment for Adult Lower Limb Spasticity
Source: PM R. 2021 Jan 11;13(10):1079–93. doi: 10.1002/pmrj.12517 (PMC8519010; doi:10.1002/pmrj.12517)
Supplement: Supplementary file 1 — Appendix S1 Supporting information [file PMRJ-13-1079-s001.docx]

**SUPPORTING INFORMATION**

**Long-Term Observational Results from the ASPIRE Study: OnabotulinumtoxinA Treatment for Adult Lower Limb Spasticity**

**
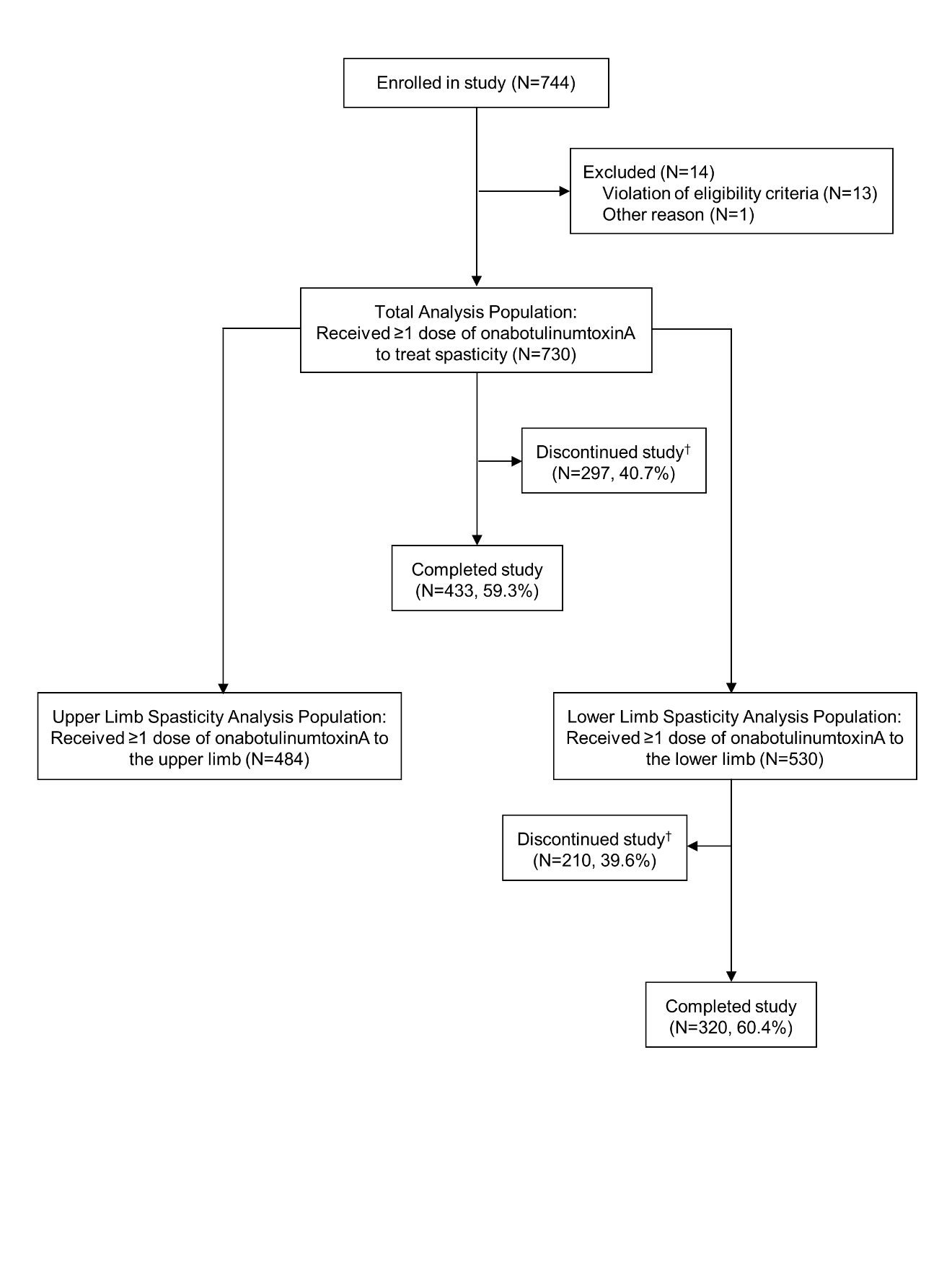
**

**Supplemental Figure 1.** Flow diagram of the ASPIRE Study Analysis. ^†^Discontinued study includes the following: all patients who failed to complete the Final Assessment form, withdrew consent, or were lost to follow-up (defined as “unable to contact after 3 attempts”). Reasons for study discontinuation were mutually exclusive. N, number of patients.

**
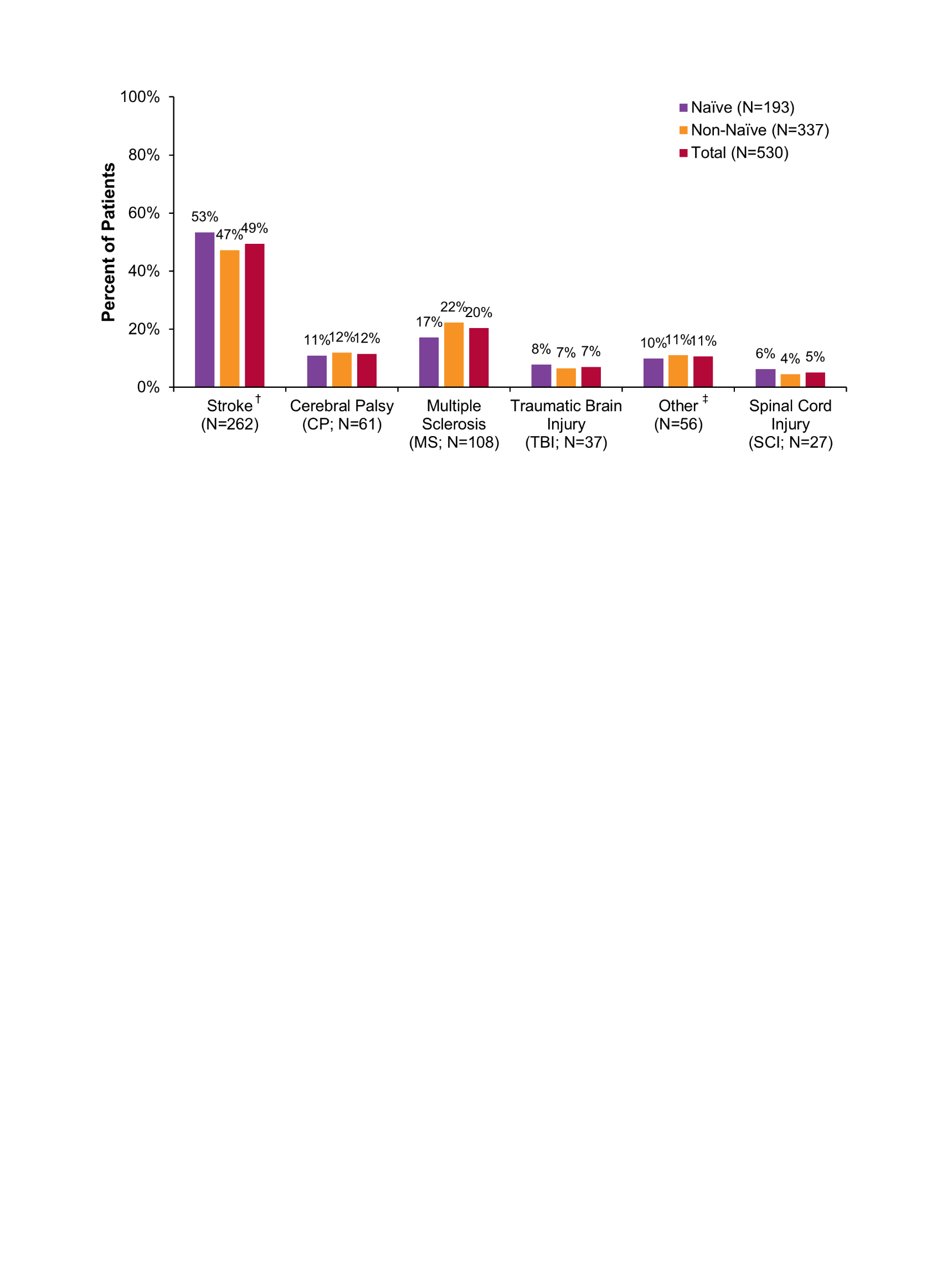
**

### **Supplemental Figure 2.** Primary Underlying Etiology of Spasticity Identified at Baseline in the Lower Limb Population. For each category (ie, naïve, non-naïve, and total), percentages sum to 100% across all etiologies. However, etiologies were not mutually exclusive, as more than one response was allowed. ^†^Stroke includes ischemic, hemorrhagic, and embolic. ^‡^Other includes hereditary spastic paraparesis, stroke during aneurysm clipping, chairi malformation, and hydrocephalus. CP, cerebral palsy; MS, multiple sclerosis; N, number of patients; TBI, traumatic brain injury; SCI, spinal cord injury.

**
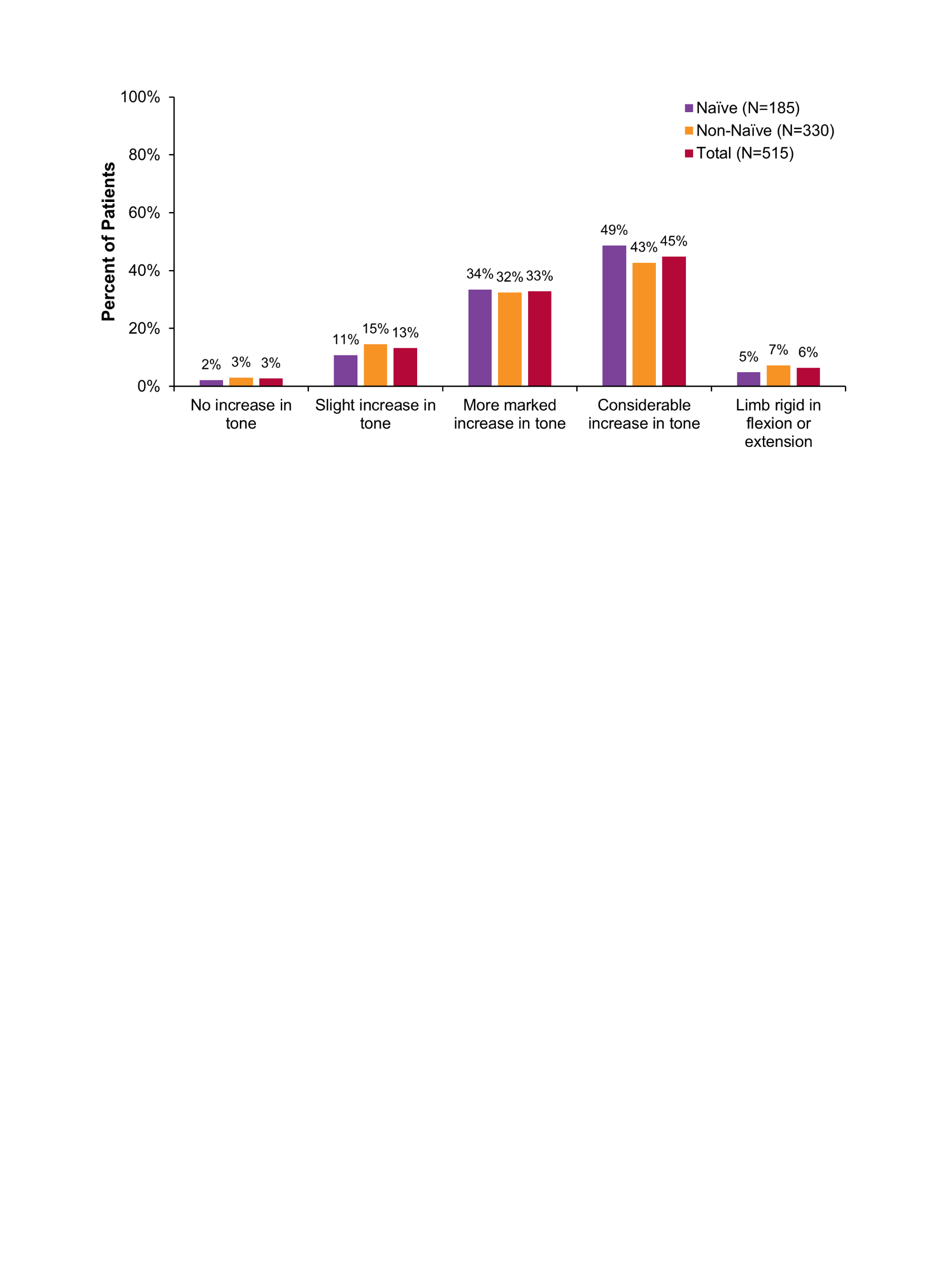
**

**Supplemental Figure 3.** Baseline Severity of Spasticity in the Lower Limb Population. At baseline, severity of spasticity was determined for each clinical presentation using the Modified Modified Ashworth Scale (MMAS [1]). Data shown here represents the mean MMAS score across all lower limb clinical presentations. For each category (ie, naïve, non-naïve, and total), percentages sum to 100% across all MMAS categories. MMAS data are missing for 15 patients (8 naïve, 7 non-naïve). N, number of patients.

**Supplemental Table 1.** Baseline Patient Demographics and Clinical Characteristics for Study Completers vs. Discontinuers in the Lower Limb Population^†^

|  | Completers  (N=320) | Discontinuers  (N=210) | Total  (N=530) |
| --- | --- | --- | --- |
| Age (years), mean (SD) | 52.2 (14.9) | 51.7 (16.2) | 52.0 (15.4) |
| BMI (kg/m^2^), mean (SD) | 26.6 (5.4) | 26.3 (5.4) | 26.4 (5.4) |
| Gender, N (%)^‡^  Female  Male | 182 (56.9)  138 (43.1) | 99 (47.1)  111 (52.9) | 281 (53.0)  249 (47.0) |
| Race, N (%)^‡^  Caucasian  Black/African/Caribbean  Asian  Latino/Hispanic  Middle Eastern/Arab  American Indian/Alaska Native  Other  Data Not Available | 273 (85.3)  27 ( 8.4)  4 ( 1.3)  4 ( 1.3)  1 ( 0.3)  1 ( 0.3)  2 ( 0.6)  8 ( 2.5) | 134 (63.8)  32 (15.2)  32 (15.2)  6 ( 2.9)  2 ( 1.0)  0 ( 0.0)  1 ( 0.5)  3 ( 1.4) | 407 (76.8)  59 (11.1)  36 ( 6.8)  10 ( 1.9)  3 ( 0.6)  1 ( 0.2)  3 ( 0.6)  11 ( 2.1) |
| Naïve to botulinum toxin(s)  for spasticity, N (%)^‡^  Yes | 93 (29.1) | 100 (47.6) | 193 (36.4) |
| Etiology of spasticity, N (%)^‡, §^  Stroke  Yes  Multiple sclerosis  Yes  Cerebral palsy  Yes  Spinal cord injury  Yes  Traumatic brain injury  Yes | 157 (49.1)  71 (22.2)  39 (12.2)  16 ( 5.0)  12 ( 3.8) | 105 (50.0)  37 (17.6)  22 (10.5)  11 ( 5.2)  25 (11.9) | 262 (49.4)  108 (20.4)  61 (11.5)  27 ( 5.1)  37 ( 7.0) |

BMI, body mass index; N, number of patients.

^†^To be labeled a study ‘completer’, patients had to meet all of the following criteria: 1) did not discontinue within the 96-week study period, 2) were not lost to follow-up, and 3) completed the Final Assessment form. Any patient that did not meet all the criteria for a study completer, was labeled a study ‘discontinuer’.

^‡^Data shown as column percents.

^§^Etiologies were not mutually exclusive, as more than one response was allowed per patient. ‘Other’ etiology not shown, which includes hereditary spastic paraparesis, stroke during aneurysm clipping, chairi malformation, and hydrocephalus.

**Supplemental Table 2.** Patient-Reported Reasons for Withdrawal of Consent from the ASPIRE Study in the Lower Limb Population^†^

| Total  (N=530) | N (% of total) |
| --- | --- |
| Withdrew consent | 120 (22.6) |
| Reason(s) for withdrawal of consent |  |
| Patient thought the treatment was ineffective | 53 (10.0) |
| Patient had difficulty paying for the onabotulinumtoxinA treatment | 26 (4.9) |
| Related to an increase in out-of-pocket expenses | 17 (3.2) |
| Related to a change or loss in health care | 15 (2.8) |
| Changed to another treatment modality | 11 (2.1) |
| Changed to another type of botulinum toxin | 11 (2.1) |
| Changed physicians | 9 (1.7) |
| Patient experienced transportation difficulties | 7 (1.3) |
| Patient thought it was inconvenient to come in for treatment visits | 7 (1.3) |
| Physician directed | 6 (1.1) |
| Spasticity improved and no longer needed botulinum toxin treatment | 6 (1.1) |
| Concerned about risks | 5 (0.9) |
| Patient moved too far away from treating physician | 4 (0.8) |
| Side effect or other health problem | 2 (0.4) |
| Patient thought the injections were too painful | 2 (0.4) |
| Other reason(s)^‡^ | 24 (4.5) |

N, number of patients.

^†^More than one reason for discontinuation could have been selected, as categories are not mutually exclusive.

^‡^The most common “other reason()” for discontinuation included death of patient, protocol violations, site closure, and administrative issues.

**Supplemental Table 3.** Numeric Pain Rating Scale (NPRS) Following OnabotulinumtoxinA Treatment for Spasticity in the Lower Limb Population^†^

|  | Baseline  (N=530) | Tx1  (N=479) | Tx2  (N=417) | Tx3  (N=353) | Tx4  (N=303) | Tx5  (N=230) | Tx6  (N=165) | Tx7  (N=120) | Tx8  (N=38) |
| --- | --- | --- | --- | --- | --- | --- | --- | --- | --- |
| NPRS score  N (missing)^‡^  Mean (SD)  P-value^§^ | 515 (15)  4.0 (3.2) | 140 (339)  -0.7 (3.0)  0.0053 | 155 (262)  -1.2 (3.1)  <.0001 | 169 (184)  -1.4 (3.2)  <.0001 | 161 (142)  -0.8 (3.2)  0.0022 | 146 (84)  -1.1 (3.2)  <.0001 | 101 (64)  -1.0 (3.0)  0.0006 | 76 (44)  -1.3 (3.5)  0.0019 | 25 (13)  -0.1 (2.9)  0.8930 |

N, number of patients; Tx, treatment session.

^†^NPRS an 11-point rating scale (range: 0-10), where “0” represents no pain and a “10” represents the worst pain imaginable, that is used to assess pain intensity [2,3]. Patient-reported NPRS data were gathered at baseline (prior to onabotulinumtoxinA treatment), as well as 5±1 weeks post-treatment via phone or web. The mean change in NPRS scores versus baseline are shown.

^‡^Missing indicates the number of patients that did not report NPRS data following onabotulinumtoxinA administration at each treatment session.

^§^Data were analyzed using paired t-tests, with Bonferroni correction applied. Unadjusted P-values are shown in the table. A statistically significant change from baseline was accepted at *P* < .006.

**Supplemental Table 4.** Patient Satisfaction with OnabotulinumtoxinA for the Treatment of Spasticity in the Lower Limb Population^†^

|  | Tx1  (n=479) | Tx2  (n=417) | Tx3  (n=353) | Tx4  (n=303) | Tx5  (n=230) | Tx6  (n=165) | Tx7  (n=120) | Tx8  (n=38) | Overall  (n=2105) |
| --- | --- | --- | --- | --- | --- | --- | --- | --- | --- |
| (A) OnabotulinumtoxinA Tx Helped  Your Spasticity, %  n (missing)^‡^  Extremely Dissatisfied/Definitely Not  Dissatisfied/Probably Not  Neither Satisfied Nor Dissatisfied/Undecided  Satisfied/Probably Yes  Extremely Satisfied/Yes, Definitely | 134 (345)  0.7  10.4  14.9  51.5  22.4 | 144 (273)  1.4  6.3  7.6  60.4  24.3 | 159 (194)  1.9  4.4  7.5  57.2  28.9 | 149 (154)  2.0  4.0  10.7  54.4  28.9 | 130 (100)  0.0  2.3  5.4  57.7  34.6 | 91 (74)  0.0  6.6  12.1  48.4  33.0 | 74 (46)  0.0  4.1  5.4  56.8  33.8 | 24 (14)  0.0  0.0  12.5  50.0  37.5 | 905 (1200)  1.0  5.3  9.3  55.4  29.1 |
| (B) OnabotulinumtoxinA Tx Helped Spasticity  in Untreated Areas, %  n (missing)  Extremely Dissatisfied/Definitely Not  Dissatisfied/Probably Not  Neither Satisfied Nor Dissatisfied/Undecided  Satisfied/Probably Yes  Extremely Satisfied/Yes, Definitely | 81 (398)  0.0  11.1  33.3  43.2  12.3 | 81 (336)  1.2  7.4  30.9  39.5  21.0 | 95 (258)  1.1  10.5  23.2  50.5  14.7 | 102 (201)  2.9  6.9  32.4  49.0  8.8 | 79 (151)  0.0  3.8  32.9  43.0  20.3 | 55 (110)  0.0  7.3  34.5  49.1  9.1 | 49 (71)  0.0  0.0  36.7  40.8  22.4 | 16 (22)  0.0  6.3  25.0  50.0  18.8 | 558 (1547)  0.9  7.2  31.2  45.5  15.2 |
| (C) OnabotulinumtoxinA Tx Helped Your  Spasticity-Related Pain, %  n (missing)  Extremely Dissatisfied/Definitely Not  Dissatisfied/Probably Not  Neither Satisfied Nor Dissatisfied/Undecided  Satisfied/Probably Yes  Extremely Satisfied/Yes, Definitely | 106 (373)  0.9  6.6  14.2  53.8  24.5 | 115 (302)  1.7  7.0  7.8  58.3  25.2 | 135 (218)  2.2  3.7  7.4  61.5  25.2 | 121 (182)  1.7  5.8  11.6  52.9  28.1 | 109 (121)  0.0  0.9  8.3  57.8  33.0 | 78 (87)  0.0  2.6  7.7  51.3  38.5 | 60 (60)  1.7  3.3  8.3  50.0  36.7 | 19 (19)  0.0  0.0  15.8  68.4  15.8 | 743 (1362)  1.2  4.3  9.6  56.1  28.8 |
| (D) Satisfaction with How Fast You Felt OnabotulinumtoxinA Tx Working, %  n (missing)  Extremely Dissatisfied/Definitely Not  Dissatisfied/Probably Not  Neither Satisfied Nor Dissatisfied/Undecided  Satisfied/Probably Yes  Extremely Satisfied/Yes, Definitely | 133 (346)  0.8  12.0  18.0  41.4  27.8 | 144 (273)  2.8  6.9  12.5  52.8  25.0 | 159 (194)  1.3  3.1  11.9  54.1  29.6 | 148 (155)  2.0  6.1  10.8  54.1  27.0 | 132 (98)  0.0  3.8  4.5  55.3  36.4 | 90 (75)  0.0  5.6  6.7  53.3  34.4 | 73 (47)  0.0  2.7  9.6  54.8  32.9 | 24 (14)  0.0  4.2  4.2  54.2  37.5 | 903 (1202)  1.1  5.9  10.7  52.2  30.1 |
| (E) Satisfaction with How Long You Felt OnabotulinumtoxinA Tx Working, %  n (missing)  Extremely Dissatisfied/Definitely Not  Dissatisfied/Probably Not  Neither Satisfied Nor Dissatisfied/Undecided  Satisfied/Probably Yes  Extremely Satisfied/Yes, Definitely | 134 (345)  0.7  12.7  17.2  49.3  20.1 | 143 (274)  2.1  11.2  12.6  53.8  20.3 | 156 (197)  2.6  12.2  12.2  55.1  17.9 | 148 (155)  2.7  8.8  9.5  59.5  19.6 | 132 (98)  0.0  9.1  9.1  62.1  19.7 | 91 (74)  0.0  7.7  14.3  57.1  20.9 | 72 (48)  2.8  8.3  16.7  51.4  20.8 | 24 (14)  0.0  8.3  8.3  62.5  20.8 | 900 (1205)  1.6  10.2  12.6  55.9  19.8 |
| (F) OnabotulinumtoxinA Tx Helped You Do  Things Around the House, %  n (missing)  Extremely Dissatisfied/Definitely Not  Dissatisfied/Probably Not  Neither Satisfied Nor Dissatisfied/Undecided  Satisfied/Probably Yes  Extremely Satisfied/Yes, Definitely | 133 (346)  3.0  10.5  33.8  37.6  15.0 | 142 (275)  1.4  11.3  23.9  47.9  15.5 | 158 (195)  2.5  8.9  30.4  42.4  15.8 | 145 (158)  2.8  8.3  28.3  43.4  17.2 | 128 (102)  0.0  7.8  27.3  49.2  15.6 | 87 (78)  0.0  14.9  20.7  47.1  17.2 | 73 (47)  1.4  6.8  28.8  46.6  16.4 | 24 (14)  0.0  12.5  37.5  41.7  8.3 | 890 (1215)  1.7  9.8  28.2  44.5  15.8 |
| (G) OnabotulinumtoxinA Tx Helped You  Participate in Social Activities, %  n (missing)  Extremely Dissatisfied/Definitely Not  Dissatisfied/Probably Not  Neither Satisfied Nor Dissatisfied/Undecided  Satisfied/Probably Yes  Extremely Satisfied/Yes, Definitely | 132 (347)  1.5  11.4  28.0  45.5  13.6 | 145 (272)  1.4  11.7  21.4  49.0  16.6 | 155 (198)  1.3  9.0  22.6  51.0  16.1 | 148 (155)  2.7  9.5  20.9  48.0  18.9 | 128 (102)  0.8  5.5  19.5  55.5  18.8 | 89 (76)  1.1  12.4  18.0  48.3  20.2 | 74 (46)  0.0  4.1  31.1  43.2  21.6 | 24 (14)  0.0  12.5  33.3  41.7  12.5 | 895 (1210)  1.3  9.4  23.0  48.8  17.4 |
| (H) OnabotulinumtoxinA Tx Helped You  Participate in Therapy/Exercise, %  n (missing)  Extremely Dissatisfied/Definitely Not  Dissatisfied/Probably Not  Neither Satisfied Nor Dissatisfied/Undecided  Satisfied/Probably Yes  Extremely Satisfied/Yes, Definitely | 116 (363)  0.0  7.8  15.5  56.9  19.8 | 127 (290)  0.8  7.1  11.8  55.9  24.4 | 134 (219)  0.7  2.2  14.9  53.7  28.4 | 130 (173)  1.5  4.6  12.3  60.0  21.5 | 112 (118)  3.6  1.8  12.5  60.7  21.4 | 77 (88)  1.3  7.8  6.5  61.0  23.4 | 67 (53)  0.0  4.5  13.4  55.2  26.9 | 21 (17)  0.0  4.8  14.3  61.9  19.0 | 784 (1321)  1.1  5.0  12.8  57.7  23.5 |
| (I) Continue OnabotulinumtoxinA to Treat  Your Spasticity, %  n (missing)  Extremely Dissatisfied/Definitely Not  Dissatisfied/Probably Not  Neither Satisfied Nor Dissatisfied/Undecided  Satisfied/Probably Yes  Extremely Satisfied/Yes, Definitely | 134 (345)  0.7  2.2  10.4  16.4  70.1 | 145 (272)  0.7  0.7  9.7  17.9  71.0 | 159 (194)  1.3  0.6  6.3  18.9  73.0 | 149 (154)  0.0  2.0  6.0  18.1  73.8 | 132 (98)  0.0  0.8  5.3  18.9  75.0 | 91 (74)  0.0  0.0  6.6  12.1  81.3 | 74 (46)  0.0  0.0  2.7  14.9  82.4 | 24 (14)  0.0  0.0  4.2  20.8  75.0 | 908 (1197)  0.4  1.0  6.9  17.3  74.3 |

n, number of treatment sessions; Tx, treatment session.

^†^At 5±1 weeks post-treatment, patient satisfaction with onabotulinumtoxinA (referred to as BOTOX in the case report form) treatment was collected via phone or web. For questions B, C, and H, the percentage of treatment sessions was recalculated to exclude those in which patients indicated that the question was “not applicable”. The number of treatment sessions in which “not applicable” was selected is included in the missing data. Unless indicated, data are presented as percent of treatment sessions.

^‡^Missing indicates the number of patients that did not complete the patient satisfaction questionnaire following onabotulinumtoxinA administration at each treatment session.

**Supplemental Table 5.** Adverse Events and Serious Adverse Events Reported in >1% of Patients in the Lower Limb Population in the ASPIRE Study

|  | Patients, N (%) | Events, n |
| --- | --- | --- |
| AEs  Fall  Muscular weakness  Urinary tract infection  Back pain  Upper respiratory tract infection  Pain in extremity  Depression  Multiple sclerosis relapse  Peripheral edema  Muscle spasticity  Hypertension  Constipation  Bronchitis  Musculoskeletal pain  Cellulitis  Arthralgia  Pneumonia  Asthenia  Headache  Seizure  Gastroesophageal reflux disease  Dizziness | 33 (6.2)  17 (3.2)  14 (2.6)  12 (2.3)  12 (2.3)  10 (1.9)  10 (1.9)  9 (1.7)  9 (1.7)  8 (1.5)  8 (1.5)  8 (1.5)  8 (1.5)  8 (1.5)  7 (1.3)  7 (1.3)  7 (1.3)  7 (1.3)  6 (1.1)  6 (1.1)  6 (1.1)  6 (1.1) | 45  17  21  14  13  10  10  11  9  10  9  9  9  8  9  9  7  7  7  6  6  6 |
| SAEs  Urinary tract infection | 6 (1.1) | 8 |

AE, adverse events; n, number of AEs; N, number of patients; SAE, serious AEs.

**References**

1. Abolhasani H, Ansari NN, Naghdi S, Mansouri K, Ghotbi N, Hasson S. Comparing the validity of the Modified Modified Ashworth Scale (MMAS) and the Modified Tardieu Scale (MTS) in the assessment of wrist flexor spasticity in patients with stroke: protocol for a neurophysiological study. BMJ Open 2012; 2(6).

2. Farrar JT, Polomano RC, Berlin JA, Strom BL. A comparison of change in the 0-10 numeric rating scale to a pain relief scale and global medication performance scale in a short-term clinical trial of breakthrough pain intensity. Anesthesiology 2010; 112(6):1464-1472.

3. Farrar JT, Young JP, Jr., LaMoreaux L, Werth JL, Poole RM. Clinical importance of changes in chronic pain intensity measured on an 11-point numerical pain rating scale. Pain 2001; 94(2):149-158.
